# A prospective, randomized assessment of a novel, local antibiotic releasing platform for the prevention of superficial and deep surgical site infections

**DOI:** 10.1007/s10151-022-02693-y

**Published:** 2022-09-01

**Authors:** O. Zmora, Y. Stark, O. Belotserkovsky, M. Reichert, G. A. Kozloski, N. Wasserberg, H. Tulchinsky, L. Segev, A. J. Senagore, N. Emanuel

**Affiliations:** 1 Shamir Medical Center, Be’er Ya’akov, Israel; 2 PolyPid Ltd, Petach Tikvah, Israel; 3 Rabin Medical Center, Beilinson Campus, Petach Tikva, Israel; 4 Tel-Aviv Sourasky Medical Center, Tel Aviv, Israel; 5 Sheba Medical Center, Tel-Hashomer, Ramat Gan, Israel

**Keywords:** Surgical site infection, Localized antibiotic therapy, Doxycycline

## Abstract

**Background:**

Despite significant advances in infection control guidelines and practices, surgical site infections (SSIs) remain a substantial cause of morbidity, prolonged hospitalization, and mortality among patients having both elective and emergent surgeries. D-PLEX_100_ is a novel, antibiotic-eluting polymer–lipid matrix that supplies a high, local concentration of doxycycline for the prevention of superficial and deep SSIs. The aim of our study was to evaluate the safety and efficacy of D-PLEX in addition to standard of care (SOC) in preventing superficial and deep surgical site infections for patients undergoing elective colorectal surgery.

**Methods:**

From October 10, 2018 to October 6, 2019, as part of a Phase 2 clinical trial, we randomly assigned 202 patients who had scheduled elective colorectal surgery to receive either standard of care SSI prophylaxis or D-PLEX_100_ in addition to standard of care. The primary objective was to assess the efficacy of D-PLEX_100_ in superficial and deep SSI reduction, as measured by the incidence of SSIs within 30 days, as adjudicated by both an individual assessor and a three-person endpoint adjudication committee, all of whom were blinded to study-group assignments. Safety was assessed by the stratification and incidence of treatment-emergent adverse events.

**Results:**

One hundred and seventy-nine patients were evaluated in the per protocol population, 88 in the intervention arm [51 males, 37 females, median age (64.0 range: 19–92) years] and 91 in the control arm [57 males, 34 females, median age 64.5 (range: 21–88) years]. The SSI rate within 30 day post-index surgery revealed a 64% relative risk reduction in SSI rate in the D-PLEX_100_ plus standard of care (SOC) group [*n* = 7/88 (8%)] vs SOC alone [*n* = 20/91 (22%)]; *p* = 0.0115. There was no significant difference in treatment-emergent adverse events.

**Conclusions:**

D-PLEX_100_ application leads to a statistically significant reduction in superficial and deep surgical site infections in this colorectal clinical model without any associated increase in adverse events.

## Introduction

Despite advances in surgical technique, adoption of procedural guidelines, increased treatment options, and better understanding of surgical wound microenvironments, surgical site infections (SSIs) remain a significant complication, and at 42.4% of all healthcare-associated infections (HAIs), have emerged as the most common HAI in the United States [1]. SSIs are the single most frequently cited reason for unplanned readmission following surgery, accounting for 19.5% of all readmission reasons across major surgical procedures and nearly 26% after colorectal surgery specifically [2].

While many efforts have been made to eliminate SSIs, up to 60% of SSIs are still considered potentially preventable with standard of care (SOC) measures that include systemic antibiotic prophylaxis [3, 4]. This may be due to issues limiting the timing and duration of target tissue penetration of SOC systemic prophylactic antibiotics. As *Sheikh *et al. note, “In clinical practice, plasma antibiotic concentrations are used as a surrogate marker for pharmacologic effect. However, for predicting therapeutic efficacy, the tissue concentration of antibiotic is more important than the plasma concentrations” [5]. Given that systematically measured concentrations are typically higher than those found in the subcutaneous tissues surrounding and within the wound bed [6], particularly for patients with obesity, diabetes, and peripheral vascular disease [7], low loco-regional tissue penetration, and hence duration of effect, is likely a significant and overlooked issue in SSI prophylaxis measures.

**Fig. 1:**
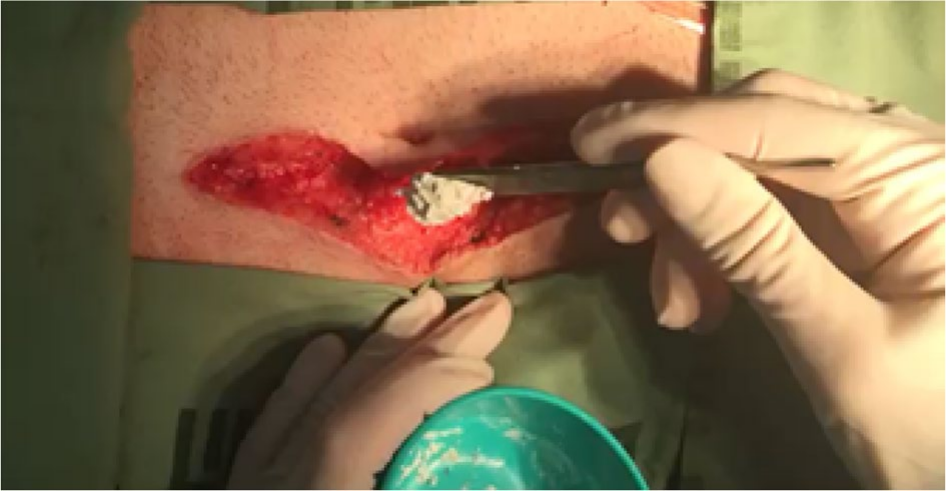
D-PLEX100 being applied to the incision edges at closure

D-PLEX_100_ (D-PLEX) (PolyPid Ltd., Petach Tikvah, Israel) is a locally applied doxycycline formulation which pairs an innovative Polymer-Lipid Encapsulation matriX (PLEX) platform with the broad-spectrum antibiotic, doxycycline and is applied to the soft-tissue wound surfaces following fascial closure prior to skin closure (Fig. 1) for the prevention of superficial and deep SSIs. This PLEX platform contains a matrix of alternating layers of polymers and lipids, which forms a protected reservoir and enables a localized, continuous release of doxycycline for a period of 30 days [8, 9]. Here, we report a Phase 2 multi-center single-blind study evaluating the safety and efficacy of D-PLEX in addition to SOC in preventing superficial and deep surgical site infections for patients who have elective colorectal surgery.

## Materials and methods

### Patients

The trial was conducted in 8 medical centers in Israel with each center supervised by a principal investigator. Eligible patients were adults, 18 years and older, undergoing elective colorectal surgery. Female patients of childbearing age were required to have a negative serum pregnancy test prior to the procedure. Patients planned for a laparoscopic approach were included if a 5 cm or greater incision was performed as part of the procedure and/or as a specimen extraction site. Key exclusion criteria included patients who were scheduled for emergency surgery or who had received doxycycline within the 4 weeks prior to screening. Patients undergoing concomitant surgical procedures via the same incision(s) were included pending consultation and approval of the site sponsor. Patients who had received neoadjuvant radiation to the abdominal area or systemic chemotherapy within 4 weeks of surgery were excluded. Patients with known hypersensitivity to doxycycline and/or the tetracycline family of drugs, to D-PLEX excipients, or who had allergies to more than 3 substances as determined from the screening questionnaire were also excluded.

### D-PLEX

D-PLEX is a new formulation of extended-release doxycycline, consisting of doxycycline, biodegradable polymers, and synthetic phospholipids along a beta-tricalcium phosphate backbone. Each 5 g D-PLEX vial contains 54.6 mg doxycycline. D-PLEX is supplied as a sterile powder and reconstituted with normal saline into a paste in the operating room. D-PLEX is administered as a single application, and the active material is continuously released for approximately 30 days.

### Study design

The study protocol was reviewed and approved by the institutional review board at each participating site, and was implemented following the principles of Good Clinical Practice and the Declaration of Helsinki, and in accordance with International Council of Harmonization guidelines and local regulations before enrollment of participants began. All patients provided written informed consent prior to any study procedures. The study was registered at the clinicaltrials.gov: NCT03633123. The patients were randomized to receive D-PLEX administered along with SOC or the SOC control arm. The prophylactic antibiotic SOC treatment, based on the Israel Ministry of Health (IMOH) guidelines and standardized for all participating sites, included a first- or second-generation cephalosporin plus metronidazole administered intravenously within 30–60 min prior to surgery. Mechanical bowel preparation (MBP) was at the discretion of the surgeon. No oral antibiotic bowel preparation (OABP) was given to either arm. For patients randomized to the treatment arm, at the time of fascia, closure D-PLEX was applied along the entire length of the surgical wound, inclusive of the fascial suture line and soft tissues of the abdominal wall, subcutaneous fat, and dermis. The D-PLEX dose was determined based on the length of the surgical incision: a 5–10 cm incision received 5 g D-PLEX, an 11–20 cm incision received 10 g D-PLEX, and an incision ≥ 21 cm received 15 g D-PLEX.

### Analysis and outcomes

Patients were randomized to either the SOC or D-PLEX plus SOC in a 1:1 ratio at day 0 via an interactive web randomization system integrated with an electronic case record form (eCRF) based on the patient’s sex, age (18–40, 41–65, and above 66), and if there was a planned ostomy creation. Patients were blinded to their study designation. Endpoints assessed included 30 day superficial and deep SSI and treatment-emergent adverse events (TEAEs). Other data collection and analyses, including pharmacokinetic data, wound microbiome, and post-operative organism colonization, were collected on varying populations and will be the discussion of a separate paper.

The incisional site was assessed at post-operative days 1, 5, 14, 30, and 60 by a blinded assessor and blinded endpoint adjudication committee (EAC). The EAC was composed of 3 physicians: 2 of whom were surgeons with expertise in colorectal surgeries and the other was an infectious diseases expert. EAC members were responsible for independently reviewing the data for each suspected SSI event and to determine whether it met the efficacy event criteria. In the event of a dispute between the blinded assessor and the committee, the committee’s adjudication prevailed. SSIs were classified following the National Healthcare Safety Network and Centers for Disease Control and Prevention Surgical Site Infection Event Reporting Manual as Superficial SSI, where the infection involved the skin and subcutaneous tissues (and not including cellulitis or stitch abscess alone) or deep SSI, when the infection involved the fascial and/or muscle layers. Organ/organ space SSIs (e.g., an intra-abdominal abscess or anastomotic leak) were assessed as TEAEs rather than SSI endpoints.

### Statistical analysis

The study planned to enroll 200 patients, with 100 subjects allocated to each treatment group. This sample size was determined to provide adequate initial data for evaluating the study objectives to provide exploratory data which would form the basis of the power calculations for planned pivotal Phase 3 trials. However, exploratory sample size calculations show that a sample of 200 subjects provides 80% power to detect an 80% decrease in the SSI rate (15% versus 3%) at a two-sided *α* = 0.10 level of significance.

Analysis consisted of summarizing the efficacy and safety data. *p* Values were based on a two-sided pooled *T* test of difference in treatment means or a two-sided Fisher’s exact test of difference in treatment proportions. *p* Values of < 0.05 are considered statistically significant. All calculations were made on the combined results of all centers, and there was no selective pooling of study centers for analyses. The data analyses were conducted using SAS^®^ Software, version 9.4 or later.

## Results

**Fig. 2:**
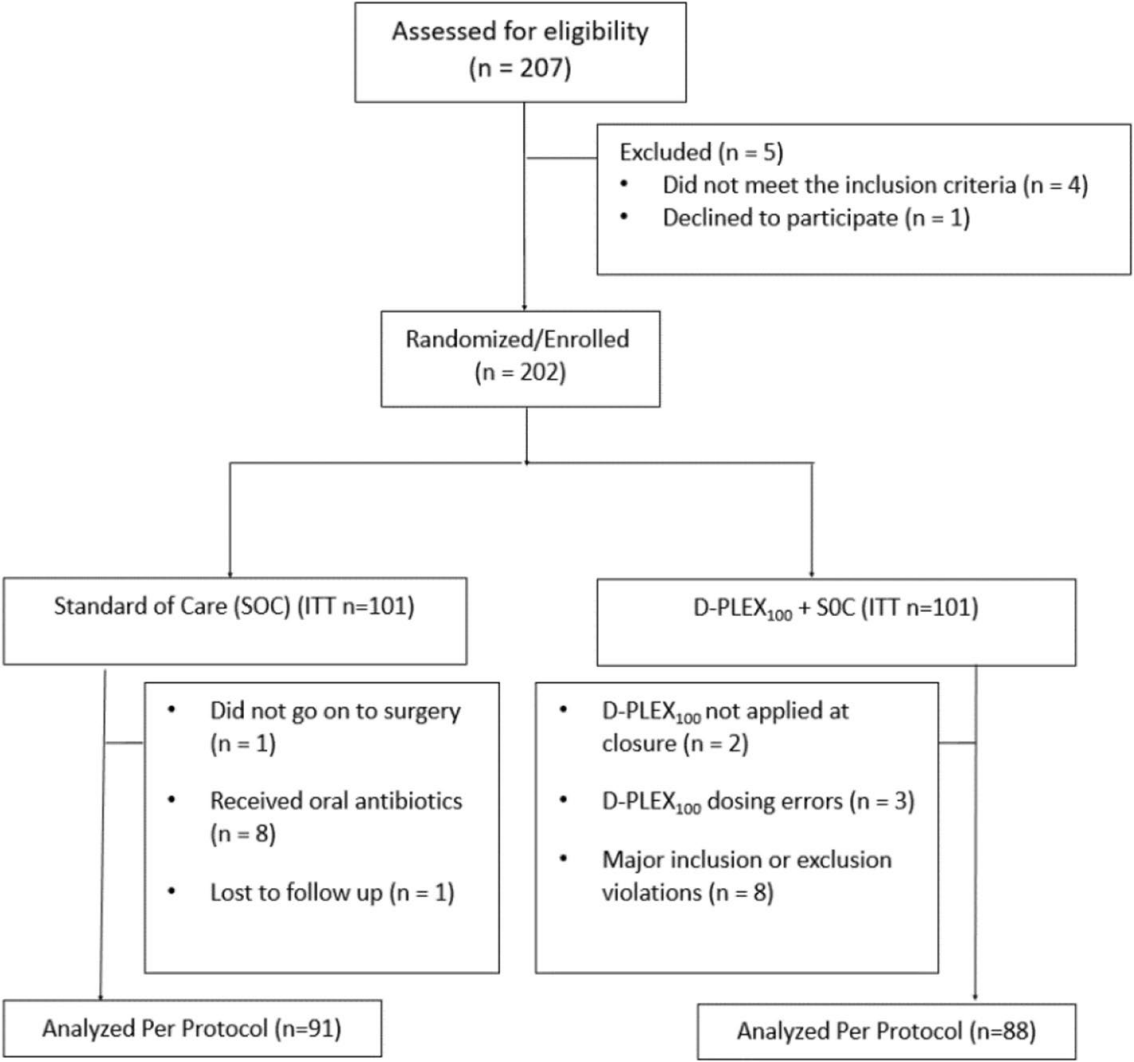
Flow Diagram of Patient Participants. *SOC* standard of care, *ITT* intention to treat

From October 2018 to October 2019, 207 patients were screened and 202 proceeded for randomization to either the SOC arm (*n* = 101) or the D-PLEX arm (*n* = 101). Analyses reported here were performed on the per protocol population (Fig. 2). In the SOC arm, 1 patient did not proceed to surgery, 8 received preoperative oral antibiotics, and 1 was lost to follow-up. In the D-PLEX arm, 2 patients did not have D-PLEX applied at closure, 3 had dosing errors, and 8 patients were found to have other major inclusion or exclusion violations. One hundred and seventy-nine patients were evaluated in the per protocol population, 88 in the intervention arm [51 males, 37 females, median age 64.0 (range: 19–92) years] and 91 in the control arm [57 males, 34 females, median age 64.5 (range: 21–88) years].

The groups were stratified based on patient demographics and surgical considerations as described in Table 1. There were no clinically meaningful differences in individual baseline characteristics between treatment arms. The only statistically significant difference between baseline characteristics was average patient height. Of note, the “Reason for Surgery” was not collected for 2 patients in the SOC arm. All surgical wounds were classified as clean-contaminated. All incisions were closed via primary intention.

**Table 1:** Patients’ demographics and baseline characteristics (per protocol population *n* = 179)

Characteristics	Treatment Arms	
	D-PLEX + SOC (*n* = 88)	SOC (*n* = 91)
Age (years), median (range)	64.0 (19–92)	64.5 (21–88)
Weight (kg), mean ± SD	75.3 ± 15.9	80.1 ± 19.4
Height (cm), mean ± SD	167.7 ± 9.7	170.7 ± 9.2
Body mass index, kg/m^2^	26.7 ± 5.3	27.3 ± 5.4
Sex (male), *n* (%)	51 (58.0)	57 (62.6)
Ethnic origin white, *n* (%)	88 (100.0)	90 (98.9)
Length of surgical incision, *n* (%)		
5–10 cm	60 (68.2)	64 (70.3)
11–20 cm	15 (17.0)	13 (14.3)
> 21 cm	13 (14.8)	14 (15.4)
Surgery included colostomy/ileostomy, *n* (%)	20 (22.7)	21 (23.1)
Reasons for surgery, *n* (%)		
Neoplasm	71 (80.7)	70 (78.7)
Inflammatory bowel disease	15 (17.0)	13 (14.6)
Other^1^	2 (2.3)	6 (6.7)
Duration of surgery( hours), mean ± SD	3.4 ± 1.4	3.3 ± 1.5
Comorbid conditions, *n* (%)		
Diabetes	25 (28.4)	22 (24.2)
Chronic obstructive pulmonary disease/smoking	16 (18.2)	16 (17.6)
Obesity/overweight	23 (26.1)	28 (30.8)
Hypertension	42 (47.7)	41 (45.1)
Peripheral vascular disease	1 (1.1)	2 (2.2)

*SOC* standard of care

^1^Other reasons for surgery: diverticular disease, volvulus

The rate of superficial and deep SSIs within 30 day post-index surgery revealed a 64% relative risk reduction in the D-PLEX cohort [*N* = 7/88 (8%)] vs. SOC [*N* = 20/91 (22%)]; *p* = 0.0115 (Table 2).

**Table 2:** Occurrence of surgical site infections (SSI) through post-operative day 30

Endpoint	D-PLEX + SOC *N* = 88	SOC *N* = 91	*p* value
SSI infection rate, *n* (%): number of patients with ≥ 1 SSI	7 (8)	20 (22)	0.0115

Data are the number (%) of patients with SSI events

*P* value based on a two-sided Fisher’s exact test of differences in treatment proportions

*SOC* standard of care

Table 3 summarizes the findings related to TEAEs. There was no statistically significant difference in incidence of TEAEs between the two groups. This includes differences in severity and incidence of serious TEAEs, although the D-PLEX cohort had a lower incidence of maximum TEAE severity which closely approached statistical significance. No TEAEs in the D-PLEX arm were deemed related to the study drug by the blinded assessor or endpoint adjudication committee. Interestingly, given that the assessors were blinded each patients’ randomization status, 15 patients (26.3%) in the SOC group had TEAEs which were deemed either “Unlikely” or “Possibly” related to the study drug (which the patients had not received).

**Table 3:** Patients with treatment-emergent adverse events (TEAEs)

Adverse event category	D-PLEX + SOC (*N* = 88)	SOC (*N* = 91)	Overall (*N* = 179)	*p* value
Subjects with at least one TEAE	51 (58.0%)	57 (62.6%)	108 (60.3%)	0.5442
Maximum TEAE severity grade				0.0512
Mild	42 (82.4%)	35 (61.4%)	77 (71.3%)	
Moderate	5 (9.8%)	10 (17.5%)	15 (13.9%)	
Severe	4 (7.8%)	12 (21.1%)	16 (14.8%)	
Relationship of TEAE to study drug				0.6684
Not related	39 (76.5%)	42 (73.7%)	81 (75.0%)	
Unlikely related	9 (17.6%)	13 (22.8%)	22 (20.4%)	
Possibly related	3 (5.9%)	2 (3.5%)	5 (4.6%)	
Related	0 (0.0%)	0 (0.0%)	0 (0.0%)	
Subjects with at least one serious TEAE	11 (12.5%)	19 (20.9%)	30 (16.8%)	0.1626

*SOC* standard of care

## Discussion

Even with the impressive advances in surgical technique and comprehensive strategies for perioperative infection reduction, superficial and deep SSIs remain a significant problem with major implications for patient outcomes, hospital metrics, and healthcare economics. Additionally, upwards of 50% of post-operative infections following colorectal surgery present outside of the hospital in the first 30 days [10], which may have substantial implications for post-operative care planning and validate the need for new, protective strategies against the development of these SSIs.

Our results show a statistically significant reduction in 30 day superficial and deep SSI incidence following D-PLEX administration without an associated increase in incidence or severity of adverse events.

It is important to consider that the safety and efficacy of D-PLEX was assessed in a setting that did not require MBP and specifically excluded OABP. While many surgeries requiring abdominal soft-tissue incisions such as urologic, gynecologic, and hepatobiliary procedures rely solely on intravenous antibiotics for systemic prophylaxis, the perceived rationale for inclusion of OABP and MBP in colorectal surgery is to reduce the potential soft-tissue wound contamination from bacteria originating in the lumen of the manipulated open bowel during the operative procedure [11–15]. Multiple clinical studies and meta-analyses have shown that MBP alone does not reduce the incidence of superficial or deep SSIs, and much of the recent literature seems to coalesce on the use of oral antibiotic prophylaxis in combination with intravenous antibiotic prophylaxis and/or mechanical bowel preparation [11, 16–20]. Despite this, there are still studies which conclude that there is no difference in outcomes with bowel preparation compared to no bowel preparation [15], even while there is ongoing disagreement on the most appropriate OABP regimen [16] and many developed countries do not routinely include OABP [21]. Given the debate, the data presented here may offer guidance on the potential role for D-PLEX in cases where bowel preparation is contraindicated, procedures for Class 2, 3, or 4 wounds, or in cases of unanticipated bowel resection to improve SSI prophylaxis in soft-tissue (myofascial layer, subcutaneous fat, and skin) incisions.

Multiple studies have evaluated the efficacy of locally applied antibiotic agents to the abdominal surgical wound with varying results [22]. The use of a gentamicin-collagen sponge placement following colorectal surgery was evaluated in a multi-center Phase 3 clinical trial and showed no SSI reduction compared to SOC [23]. More recently, a Phase 2b trial studied the use of a bioresorbable, modified-release gel containing a combination of vancomycin and gentamicin also failed to demonstrate a significant benefit in SSI reduction [24]. Of the postulated reasons for these findings, the authors note the need for “rethinking of the proposed drug formulation.” The study notes the duration of the gel at approximately 48 h, which is remarkably shorter than the roughly 700 h duration for D-PLEX [9]. While formally planned studies are certainly necessary for further validation, it is interesting to postulate the utility of a prolonged-eluted locally applied antibiotic treatment option such as D-PLEX as an addition to current standard prophylactic recommendations.

There were limitations in this study which deserve consideration. Given that nearly 100% of the study population identified as ethnically white, our sample population is uniformly homogeneous and not representative of a more diverse patient population. Since certain populations, particularly those of African descent, have a well-described higher propensity for keloid formation and may be more susceptible to adverse post-operative hypertrophic scar formation [25, 26], studies involving more diverse patient populations are required to ensure the consistent safety profile demonstrated in this study. While the SOC SSI rate of 22% may initially appear high, it is within commonly reported ranges in colorectal surgery [17–20, 27, 28]. Additionally, while it may not have had yielded any meaningful differences, other post-operative data points, such as glucose levels and body temperature, would have been important to collect and evaluate, as many of the current SSI bundle recommendations include maintaining euglycemia and normothermia [4, 27]. Although the association of SSIs with laparotomy incision type, extraction incision location, and conversion from a minimally invasive approach to open surgery are areas of clinical interest, the sample size precludes any meaningful evaluation of these relationships in this study. Although it is unlikely that MBP had a meaningful effect on SSI rate, as discussed above, since this aspect of prophylaxis was not standardized, there may be tangential biases not accounted for in the study design, and even with considerations provided earlier in the discussion, many audiences, particularly in the United States, expect the evaluation of SSI rates to involve the use of OABP and MBP and, therefore, warrant consideration in future studies.

## Conclusions

The application of D-PLEX_100_ to the incisional wound bed following myofascial closure provided a statistically significant and clinically meaningful decrease in superficial and deep SSI rates without an increase in TEAEs. As such, it is a promising addition to current SSI prophylaxis bundle recommendations and is currently under evaluation in two Phase 3 clinical trials.
